# Is there a difference in women’s experiences of care with medication vs. manual vacuum aspiration abortions? Determinants of person-centered care for abortion services

**DOI:** 10.1371/journal.pone.0225333

**Published:** 2019-11-25

**Authors:** May Sudhinaraset, Amanda Landrian, Dominic Montagu, Ziporah Mugwanga

**Affiliations:** 1 Community Health Sciences, University of California Los Angeles, Jonathan and Karin Fielding School of Public Health, Los Angeles, CA, United States of America; 2 Institute for Global Health Sciences, University of California San Francisco, School of Medicine, San Francisco, CA, United States of America; 3 Marie Stopes Kenya, Nairobi, Kenya; Johns Hopkins University Bloomberg School of Public Health, UNITED STATES

## Abstract

Little evidence exists on women’s experiences of care during abortion care, partly due to limitations in existing measures. Moreover, globally, the development and rapid growth in the availability of medication abortions (MA) has radically changed the options for safe abortions for women. It is therefore important to understand how women’s experiences of care may differ across medication and manual vacuum aspiration (MVA) abortions. This study uses a validated person-centered abortion care scale (categorized as low, medium, and high levels, with high levels representing the greatest level of person-centered care) to assess women’s experiences of care undergoing medication abortions vs. MVA. This paper reports on a cross-sectional study of 353 women undergoing abortions at one of six family planning clinics in Nairobi County, Kenya in 2018. Comparing abortion types, we found that the MVA sample was more likely to report “high” levels of person-centered abortion care compared to the MA sample (36.3% vs. 23.0%, p = 0.005). No differences were detected with respect to Respectful and Supportive Care; however, the MVA sample was significantly more likely to report “high” levels of Communication and Autonomy compared to the MA sample (23.6% vs. 11.2%, p<0.0001). In multivariable ordered logistic regression, we found that the MVA sample had a 92% greater likelihood of reporting higher person-centered abortion care scores compared to MA clients (aOR1.92, CI: 1.17–3.17). Being employed and reporting higher self-rated health were associated with higher person-centered abortion care scores, while reporting higher levels of stigma were associated with lower person-centered abortion care scores. Our findings suggest that more efforts are needed to improve the domain of Communication and Autonomy, particularly for MA clients.

## Introduction

Kenya reports high levels of unintended pregnancies. One 2015 analysis estimated that 41% of the unintended pregnancies in Kenya will end in an abortion, resulting in approximately 500,000 abortions each year [1]. The 2010 amendment of Kenya’s Constitution expanded legal abortions in cases where the health of a woman is in danger; prior to this, restrictive policies only allowed abortions to protect the woman’s life [2]. The expansion of the law to include the “health exception” has the potential to expand access to safe abortions [3]; however, ambiguity around the law, including conflicts in the 2010 Constitution, which allows abortions in certain cases, and the penal code, which criminalizes abortions, results in inconsistent implementation [4]. The result of this legal and enforcement uncertainty is that clinicians are fearful of providing abortions and patients are stigmatized [4]. In this context, while access to safe abortions exists, the quality of care for abortions remains an ongoing challenge.

Beyond clinical concerns, the experience of care for abortion recipients is often especially challenging [5]. The World Health Organization highlights experience of care and provision of care as distinct, equally important, domains of abortion quality [6]. In practice, however, patient experiential quality has been largely absent or oversimplified as “satisfaction” in much of the literature. This is problematic both because patient experience is important from a human rights perspective, and because women’s experiences can impact outcomes, adherence to post-abortion guidance, and future health-seeking decisions by the patient and women who hear of her experiences [7,8]. A systematic review found that person-centered and respectful abortion care remain a challenge globally, potentially due to deeply embedded social stigma, institutional regulations, and legal restrictions [7].

Issues with quality of care related to dignity, autonomy, privacy, social support, communication, supportive care, and the health facility environment are apparent across a variety of contexts and settings [7]. However, while recognition exists, addressing women’s experiences has been hampered by the lack of consensus on definitions of person-centered care for abortion services, and the lack of standardized measures of abortion quality, more generally [5]. In Kenya, evidence is especially sparse: most studies focused on patient experience are qualitative [9–12]. While qualitative data can identify gaps in women’s experiences of care, quantitative data is needed to understand the magnitude of the problems in quality of care, establish baseline quality of care, track improvements over time, compare quality of care across geographies and facilities, and inform quality improvements efforts.

The mix of methods for abortions makes better understanding this aspect of quality particularly relevant in Kenya. Little is known about how women’s experiences differ between medication-induced abortions (MA) and manual vacuum aspirations (MVA), two very distinct procedures. MA refers to the use of a drug or combination of drugs to terminate a pregnancy (i.e. mifepristone followed with or without misoprostol) while MVAs are a type of surgical abortion. The few studies that have assessed patient experiences across the two methods have found mixed and sometimes contradictory results pertaining to experiences of care with MA vs. MVA. For example, one quantitative study reported levels of satisfaction with response options including highly satisfied, satisfied, and not satisfied and found that there were high levels of satisfaction overall for both MA and surgical abortions [13]. The study also reported that MA clients reported higher levels of satisfaction compared to surgical abortions; however, the few women who reported dissatisfaction in the sample was higher among MA clients vs. surgical clients. This may be due to MA clients reporting higher levels of side effects such as nausea, cramping, and bleeding compared to surgical abortion clients [13]. Other quantitative studies similarly find mixed results when asking about experiences through questions pertaining to satisfaction, whether they would recommend it to others, or whether they would choose the same method again [14]. These findings are limited in that past measures used have not adequately addressed women’s experiences of specific aspects of abortion care.

The purpose of this study is to examine women’s experiences of abortion care across surgical (i.e. in this study MVA) and medication abortions in a formal health sector in Nairobi, Kenya. We surveyed women and used a validated measure of person-centered care for abortion, with two sub-domains identified including “Respectful and Supportive Care” and “Communication and Autonomy” [15]. First, this study assesses whether type of abortion is associated with person-centered care score. Second, this study compares determinants of person-centered abortion care.

## Materials and methods

### Study participants and recruitment

This is a cross-sectional study of abortion clients recruited from six family planning clinic located across Nairobi County, Kenya at Marie Stopes Kenya (MSK) facilities. Nairobi County is a metropolitan area, densely populated including slums or market centers. The six clinics were chosen based on client volume (i.e. clinics ranged from approximately 50 to 220 reproductive health clients, including abortion clients), diverse population, and interest and willingness to participate in a survey about person-centered abortion care. Abortion method is determined by gestational age and preference of the client in consultation with the provider. At MSK, approximately 70% have medication abortions and 30% MVA.

Women were eligible to participate if they: 1) received MVA or medication abortion services at the clinic the day of recruitment; 2) were at least 18 years of age; 3) spoke English or Swahili; and 4) owned a mobile phone and were comfortable being contacted by study staff via phone. We obtained written informed consent in person at the clinic from interested and eligible women prior to the initiation of study procedures. After providing consent, women were administered the study survey by a trained researcher through an electronic survey tablet, which took about 40 minutes to complete, in a private space located within the clinic. Recruitment, consent, and baseline surveys were all conducted on the same day of receiving the abortion service. We pilot tested these questions with women before launching the full survey to assess whether length of survey and survey questions were appropriate. All women who provided consent and completed the survey received mobile phone airtime equivalent to approximately $1.50 US dollars for their participation in the study.

The University of California, San Francisco (UCSF), Marie Stopes Kenya (MSK), and Innovations for Poverty Action (IPA) conducted a randomized control trial (RCT) of a post-abortion support intervention in Nairobi, Kenya in 2017. This present manuscript presents baseline data from the larger intervention study on post-abortion social support, with original plans to collect a sample of 1000 participants across three study arms (330 per study arm). The intervention included training a peer counselor and peer nurse on providing post-abortion follow up, including providing emotional and informational support to post-abortion women. All women were recruited into the study following their abortion procedure, and therefore, the intervention did not influence the type of abortion that women chose.

The sample size calculation was performed using Stata 15MP and assumed the detection of a five point difference between the intervention and control group with a power of at least 0.90, accounting for repeated measures (baseline and two follow up surveys). Only 371 women were eventually recruited into the study at baseline due to unexpected halts in service delivery relating to government bans on abortion services in Kenya at the time of the study. However, a power calculation using two-sample means test and the sample sizes across MVA vs. MA suggests sufficient sample to assess differences in means across the two abortion methods with a power of 0.76 (assuming a standard deviation of 10.24 and 10.79 from analyses).

The full study protocol and all study materials were reviewed and approved by the Ethical Review Board at the University of California, San Francisco and the Kenya Medical Research Institute.

### Measures

#### Dependent variable

The outcome of interest was the score obtained from a validated person-centered abortion care (PCAC) scale (available in S1 Table) [15], which was adapted from two other person-centered reproductive health scales [16,17]. The scale was developed using standard psychometric procedures including a review of the literature, cognitive interviews with abortion clients to determine face validity (i.e. how did they understand the question and how important was that item to them in terms of their experiences with care), expert reviews, and factor analysis to determine number of factors to include [15]. The scale includes 24 items for women receiving MVA [Cronbach’s alpha = 0.82] and 23 items for medication abortion [Cronbach’s alpha = 0.82]. One item regarding the provision of pain medication is not applicable to women receiving medication abortion. Given that most women receive the medication abortion pills at the facility (at the time of the survey), but do not typically take the pill until they are at home, women do not feel pain until they have taken the pill at home when the abortion has started. The full scale is comprised of two sub-scales. The “Respectful and Supportive Care” sub-scale includes questions such as: “Did the doctors, nurses or other staff at the facility treat you with respect?,” “Do you feel like your health information will be kept confidential at this facility?,” and “Do you think there was enough health staff in the facility to care for you?” The “Communication and Autonomy” sub-scale includes questions such as: “Did the doctors, nurses, or other health care providers call you by your name?” and “Did you feel like the doctors, nurses or other staff at the facility involved you in decisions about your abortion care?” Response options for each item ranged from 0 (“Never”) to 3 (“All of the time”) and were totaled across the 23 items which are the same for both surgical and medication abortion clients (i.e., excluding the question on pain medication) to obtain a total score ranging from 0 to 69. Higher scores indicate better PCAC. A variable was then created categorizing total PCAC scores as “low,” “medium,” or “high,” with scores in the bottom 25^th^ percentile defined as “low” and those in the top 75^th^ percentile defined as “high.” The same procedure was used to calculate and recategorize scores for each PCAC sub-scale. Sensitivity analyses were also run that included PCAC score as a continuous and binary outcome. Because the main findings did not change significantly and for ease of interpretation, we present results from the categorical outcome. We highlight results from the sensitivity analyses in the Discussion section below.

#### Independent variables

Key independent variables of interest included type of abortion procedure (MVA versus medication abortion), women’s demographic characteristics, self-rated health status, and beliefs and feelings regarding abortion. Demographic characteristics included women’s age, marital status, educational attainment, employment status, religion, and number of pregnancies (including their current pregnancy), live births, and living children. Women’s current self-rated health was assessed by asking: “How will you rate your health now, will you say it is excellent, very good, good, fair, poor, or very poor?” A binary variable was then created to capture whether a woman rated their current health as excellent, very good, or good versus poor or very poor (1 = excellent, very good, or good, 0 = poor or very poor). Ability to pay for the abortion procedure was assessed by asking: “How easy is it for you to get money to buy what you need for your procedure and pay for services at this MSK Clinic? Would you say it is very easy, easy, difficult, or very difficult?” Ability to pay for transportation to the clinic for the abortion was assessed by asking: How easy is it for you to pay for transportation to this MSK Clinic? Would you say it is very easy, easy, difficult, or very difficult?” From these items, two binary variables were created to capture whether it was easy for the woman to get money for the abortion procedure and to pay for transportation to the clinic for the abortion procedure, respectively (1 = Yes, 0 = No). Women were also asked if abortion was legal in Kenya; response options included “yes,” “no,” and “do not know.” Abortion-related stigma was assessed using three items from the “worries about judgment” domain of the Individual Level Abortion Stigma (ILAS) scale [18]. These items asked women to describe how worried they were about: other people finding out about their abortion; disappointing someone they love; or people gossiping about them. Each item had response options ranging from “not worried” to “extremely worried” and was later recoded as a dichotomous variable capturing whether women reported feeling worried about that respective scenario (1 = Yes, 0 = No).

### Data analyses

All analyses were performed using StataSE version 15.1 using descriptive, bivariate, and multivariable statistics. As missing data represented less than 1% of responses, they were recoded to the most prevalent response category; only questions on marital status, religion, ability to get money to pay for abortion procedure, the legal status of abortion in Kenya, and abortion stigma had missing data. We conducted sensitivity analyses, whereby analyses were repeated after excluding all missing data, and no findings differed.

We used Pearson’s chi-square tests to examine differences in the distribution of demographic characteristics, self-rated health status, and beliefs and feelings around abortion by abortion procedure type (MVA versus medication abortion). Pearson’s chi-square tests were also used to examine differences in the distribution of PCAC scores by abortion procedure type.

We ran bivariate and multivariable ordered logistic regressions to examine associations between type of abortion procedure, demographic characteristics, self-rated health status, and beliefs and feelings around abortion and total PCAC score. In multivariable analyses, we included variables significant in bivariate analyses (p-value<0.05); multivariable analyses also controlled for age, marital status, educational attainment, and employment status regardless of the significance in bivariate analyses. All regression analyses accounted for potential intragroup correlation at the clinic-level (i.e., that observations are independent across clinics but may not be independent within clinics) by using appropriate robust standard error procedures.

## Results

In total, 383 women were approached for the study; two were ineligible, seven refused consent for the study. Of those who consented, only three did not complete the survey and a total of 371 women completed the survey. Of these, 353 (95%) women had valid data for all PCAC scale items, including 157 who received a MVA and 196 who received medication abortion.

### Demographic characteristics, self-rated health status, and beliefs and feelings around abortion

Demographic characteristics, self-rated health status, and beliefs and feelings around abortion stratified by type of abortion procedure received are presented in Table 1. P-values corresponding to chi-square tests are also included. A higher proportion of women in the MVA sample were aged 35 years or older compared to the medication abortion sample (18% vs. 7%, respectively; p = 0.001), while a higher proportion in the medication abortion sample compared to the MVA sample were aged less than 20 years (13% vs. 4%, respectively; p = 0.001). Most women among both MVA and medication abortion samples, respectively, were not married, partnered, or cohabitating (73% and 81%), had at least a secondary education (87% and 93%), were currently employed for pay (62% and 55%), and identified as Christian (95% and 96%); no statistically significant differences were detected in these characteristics across samples. Compared to the MVA sample, a higher proportion of women in the medication abortion sample reported this to be their first pregnancy (40% vs. 54%, respectively; p = 0.03). A higher proportion of women in the MVA sample rated their current health as excellent, very good, or good (66% vs. 57%, p = 0.066), although the difference was only approaching statistical significance.

**Table 1:** Sociodemographic characteristics, self-rated health, and beliefs and feelings regarding abortion stratified by abortion procedure type.

*Characteristic*	N (%)			P-value*
	Total Sample (N = 353)	MVA (N = 157)	Medication Abortion (N = 196)	
Age, years				0.001
Less than 20	31 (8.8)	6 (3.8)	25 (12.8)	
20–24	137 (38.8)	61 (38.9)	76 (38.8)	
25–29	94 (26.6)	37 (23.6)	57 (29.1)	
30–34	50 (14.2)	25 (15.9)	25 (12.8)	
35 or older	41 (11.6)	28 (17.8)	13 (6.6)	
Married, partnered, or cohabitating	79 (22.4)	42 (26.8)	37 (18.9)	0.078
Education				0.098
Primary or less	34 (9.6)	20 (12.7)	14 (7.1)	
Secondary or vocational	143 (40.5)	56 (35.7)	87 (44.4)	
College or University	176 (49.9)	81 (51.6)	95 (48.5)	
Employed for pay	205 (58.1)	97 (61.8)	108 (55.1)	0.206
Religion				0.729
Christian	338 (95.8)	149 (94.9)	189 (96.4)	
Muslim	10 (2.8)	5 (3.2)	5 (2.6)	
None	5 (1.4)	3 (1.9)	2 (1.0)	
Number of pregnancies				0.01
1	168 (47.6)	63 (40.1)	105 (53.6)	
2	87 (24.7)	41 (26.1)	46 (23.5)	
3	47 (13.3)	25 (15.9)	22 (11.1)	
4	28 (7.9)	11 (7.0)	17 (8.7)	
5 or more	23 (6.5)	17 (10.8)	6 (3.1)	
Number of live births				0.03
0	196 (55.5)	76 (48.4)	120 (61.2)	
1	77 (21.8)	37 (23.6)	40 (20.4)	
2	48 (13.6)	23 (14.7)	25 (12.8)	
3 or more	32 (9.1)	21 (13.4)	11 (5.6)	
Number of living children				0.04
0	199 (56.4)	78 (49.7)	121 (61.7)	
1	78 (22.1)	35 (22.3)	43 (21.9)	
2	44 (12.5)	24 (15.3)	20 (10.2)	
3 or more	32 (9.1)	20 (12.7)	12 (6.1)	
Current self-rated health is excellent, very good, or good	215 (60.9)	104 (66.2)	111 (56.6)	0.066
Easy to get money for abortion procedure	97 (27.5)	45 (28.7)	52 (26.5)	0.656
Easy to pay for transportation to clinic for abortion procedure	284 (80.5)	121 (77.1)	163 (83.2)	0.151
Abortion is legal in Kenya				0.808
Yes	112 (31.7)	54 (34.4)	58 (29.6)	
No	168 (47.6)	72 (45.9)	96 (49.0)	
Do not know	73 (20.7)	31 (19.8)	42 (21.4)	
Worried other people might find out about abortion procedure	137 (38.8)	61 (38.9)	75 (38.3)	0.910
Worried about disappointing loved ones	134 (38.0)	53 (33.8)	80 (40.8)	0.174
Worried people will gossip about them	81 (23.0)	40 (25.5)	41 (20.9)	0.311

Column percentages shown; may not add to 100 due to rounding. MVA = Manual vacuum aspiration.

*Pearson chi-square test of differences between MVA and medication abortion clients.

No statistically significant differences were detected in items pertaining to beliefs and feelings around abortion across the MVA and medication abortion samples. Most women among both samples reported it was easy to pay for transportation to the clinic (77% and 83%, respectively); however, the majority said it was difficult to get money to pay for the procedure itself (66% and 57%, respectively). Nearly half of women in both samples reported abortion to be illegal in Kenya; about one-fifth reported that they did not know whether abortion was legal. About 38% of women in both samples reported feeling worried that people might find out about their abortion, while 33% of women in the MVA sample and 41% of women in the medication abortion sample reported feeling worried about disappointing loved ones. A lower proportion of women in both MVA and medication abortion samples reported feeling worried that people would gossip about them (26% and 21%, respectively). None of these characteristics were statistically significantly different by abortion procedure type.

The distribution of PCAC scores (low, medium, or high) for the full scale and each sub-scale by abortion procedure type, including the p-value corresponding to Pearson’s chi-square tests, are included in Table 2. A significantly higher proportion of women in the MVA sample had PCAC scores categorized as “high” compared to those in the medication abortion sample (36% vs. 23%, respectively; p = 0.005). While no significant differences were detected in the distribution of “Respectful and Supportive Care” sub-scale scores, a significantly higher proportion of women in the MVA sample had scores categorized as “high” in the “Communication and Autonomy” sub-scale than those in the medication abortion sample (24% vs. 11%, respectively; p<0.0001). The distributions of the “Respectful and Supportive Care” and “Communication and Autonomy” sub-scale items, stratified by abortion type, are included in S2 and S3 Tables, respectively.

**Table 2:** Comparison of PCAC scores by abortion procedure type.

*PCAC Score*	N (%)			P-value*
	Total Sample (N = 353)	MVA (N = 157)	Medication Abortion (N = 196)	
**Full PCAC Scale**				0.005
Low	79 (22.4)	25 (15.9)	54 (27.6)	
Medium	172 (48.7)	75 (47.8)	97 (49.5)	
High	102 (28.9)	57 (36.3)	45 (23.0)	
**Respectful and Supportive Care Sub-Scale**				0.478
Low	84 (23.8)	34 (21.7)	50 (25.5)	
Medium	139 (39.4)	60 (38.2)	79 (40.3)	
High	130 (36.8)	63 (36.8)	67 (34.2)	
**Communication and Autonomy Sub-Scale**				<0.0001
Low	76 (21.5)	19 (12.1)	57 (29.1)	
Medium	218 (61.8)	101 (64.3)	117 (59.7)	
High	59 (16.7)	37 (23.6)	22 (11.2)	

Column percentages shown; may not add to 100 due to rounding. PCAC = Person-centered abortion care. MVA = Manual vacuum aspiration.

*Pearson chi-square test of differences between MVA and medication abortion clients.

### Bivariate analysis

Results from bivariate ordered logistic regressions examining factors associated with PCAC score (after accounting for potential intragroup correlation at the clinic-level) are provided in Table 3 under the column labeled “Unadjusted Coeff.” Type of abortion procedure was significantly associated with PCAC score. Not accounting for other factors, the odds of having high versus low or medium PCAC scores was 95% higher among MVA clients than MA clients. Employment status, current health status, and being worried about disappointing loved ones were also found to be significantly associated with PCAC score. Specifically, the odds of having high versus low or medium PCAC score was 62% higher among women who reported working for pay than those who did not. Those who rated their current health as excellent, very good, or good had an odds of high versus low or medium PCAC score that was about 100% higher than those who did not. Compared to women who were not worried about disappointing loved ones, those who were worried had an odds of high versus low or medium PCAC score that was 37% lower. No other significant differences in PCAC score were found by age, marital status, educational attainment, parity, beliefs around the legality of abortion in Kenya, ease of paying for abortion or transportation to the clinic, and being worried about people finding out about their abortion or that people would gossip about them.

**Table 3:** Examining factors associated with PCAC score using bivariate and multivariable ordered logistic regression.

*Variables*	PCAC Score	
	Unadjusted OR (95% CI)	Adjusted OR (95% CI)
Abortion type		
Medication	Ref	Ref
MVA	1.95 (1.18, 3.24)**	1.92 (1.17, 3.17)**
Age, years		
Less than 20	Ref	Ref
20–24	0.57 (0.18, 1.78)	0.44 (0.14, 1.41)
25–29	0.64 (0.21, 1.96)	0.43 (0.13, 1.44)
30–34	1.18 (0.43, 3.24)	0.68 (0.23, 2.03)
35 or older	0.83 (0.34, 2.02)	0.39 (0.14, 1.13)
Married, partnered, or cohabitating		
No	Ref	Ref
Yes	1.18 (0.78, 1.78)	1.04 (0.54, 2.00)
Education		
Primary or less	Ref	Ref
Secondary or vocational	0.80 (0.30, 2.13)	0.87 (0.24, 3.21)
College or University	0.86 (0.34, 2.19)	0.88 (0.27, 2.81)
Employed for pay		
No	Ref	Ref
Yes	1.62 (1.13, 2.31)***	1.54 (0.99, 2.42)*
Number of births		
0	Ref	
1	1.27 (0.60, 2.68)	
2	1.32 (0.85, 2.06)	
3 or more	1.63 (0.69, 3.87)	
Current self-rated health is excellent, very good, or good		
No	Ref	Ref
Yes	2.02 (1.46, 2.78)***	1.80 (1.22, 2.66)***
Easy to get money for abortion procedure		
No	Ref	
Yes	1.22 (0.96, 1.57)	
Easy to pay for transportation to clinic for abortion procedure		
No	Ref	
Yes	1.07 (0.47, 2.40)	
Abortion is legal in Kenya		
No	Ref	
Yes	1.22 (0.77, 1.94)	
Do not know	1.13 (0.84, 1.51)	
Worried other people might find out about abortion procedure		
No	Ref	
Yes	0.92 (0.68, 1.24)	
Worried about disappointing loved ones		
No	Ref	Ref
Yes	0.63 (0.43, 0.92)**	0.71 (0.49, 1.03)*
Worried people will gossip about them		
No	Ref	
Yes	0.99 (0.69, 1.43)	

Adjusted models control for age, marital status, educational attainment, and employment status (regardless of significance in bivariate analyses), as well as any variables that were significant in bivariate analyses at p-value<0.01. OR = Odds ratio. CI = Confidence interval. Ref = Referent category. MVA = Manual vacuum aspiration.

*p-value<0.10,

**p-value<0.05,

***p-value<0.01.

### Multivariable analysis

The results of the multivariable order logistic regression analysis examining factors associated with PCAC score (after accounting for potential intragroup correlation at the clinic-level) are also provided in Table 3. Type of abortion procedure remained significant in multivariable analyses after adjusting for other factors. All else being equal, MVA clients had an odds of high versus low or medium PCAC score that was 92% higher than MA clients. Further, women who rated their current health as either excellent, very good, or good had an odds of high versus low or medium PCAC score that was 80% higher than those who did not. Employment status and being worried about disappointing loved ones were marginally significant at p-values less than 0.10; after controlling for other factors, the odds of high versus low or medium PCAC score was 55% higher among women who reported working for pay than those who did not (p = 0.06) and 29% lower among women who were worried about disappointing loved ones compared to those who were not (p = 0.07), respectively. Other demographic characteristics (age, marital status, and educational attainment) remained statistically insignificant in the multivariable analysis. Number of births and being worried about disappointing loved ones lost statistical significance in the multivariable analysis after controlling for other factors.

## Discussion

Person-centered care is a critical dimension of quality of care [6]; it is important from a human rights perspective and influencing long-term health outcomes, and one that is best assessed by the woman herself [7,8]. Because of the highly stigmatized nature of abortions, little is known about the quality of care for abortions beyond safety. To our knowledge, this study is the first to examine person-centered care for abortion services using a validated scale. Our overall findings are positive: across both methods, most women felt that they were treated with respect, facility staff cared about them, their information was kept confidential, they were given attention, there was enough staff, and they could trust the staff who were there. This is in keeping with other research: a study in Mexico found that women reported consistently high satisfaction after receiving abortions in government clinics [19]. Another study found that over 98% of women reported being satisfied with their method chosen, with medication clients more likely to choose this method again compared to surgical clients (94% vs. 78%, respectively) [20]. In the US, similarly high satisfaction rates were found for surgical abortions; closely matching satisfaction for all types of ambulatory surgical care for women [21].

Challenges around the quality of abortion experiences in Kenya nevertheless remain, particularly regarding Communication and Autonomy. In our findings, only 61% of MVA clients and 57% of medication clients reported that providers called them by their name all the time. Only 77% and 74%, respectively, of women said they were consented to care, and only 60% of MVA clients and approximately 41% of medication clients indicated that providers talked to them about how they were feeling [S2 and S3 Tables]. These are all areas of care and treatment identified by women as important markers of quality and person-centered care [8].

Bivariate and multivariate analyses suggest that abortion type is predictive of women’s person-centered care score. MVA clients were more likely to report that they were treated in a friendly manner, called by their name, asked about how they were feeling, and how much pain they were in. There are several potential explanations for these findings. First, MVA clients have more opportunities to interact with providers: more time, more communication, and potentially being present while pain is experienced at the clinic. As a result, providers may engage actively to ask women how much pain they were in and how they were feeling. Second, in Kenya, training for MVA includes a “vocal local” method that involves speaking to a woman throughout the procedure. This method is personalized, and providers are trained to call the woman by her name, an important component of PCAC. It is also plausible that expectations of care and prior experiences with healthcare systems may explain the difference in person-centered care, given that these factors may play a role in the decision to seek reproductive health services [8].

In both bivariate and multivariate regressions, other factors that were associated with higher levels of person-centered care include being employed and reporting higher self-rated health; on the other hand, being worried about disappointing loved ones, a measure of abortion stigma, was associated with lower levels of person-centered care. These findings are not surprising; for example, employment status is associated with women’s empowerment and higher status women; therefore, it is likely that women who are employed may be more likely to be able to advocate for themselves and demand better care while providers may also treat them with more respect given their social status [7]. Moreover, women who report better health may have less anxiety about their health in general, and as such, may report a better experience [22]. We know, for example, that self-rated health has strong associations with other factors such as mortality [23] and social resources [24]. Reports of greater levels of stigma, on the other hand, was associated with lower person-centered care. This is an important finding given that abortions continue to be highly stigmatizing in Kenya [25], with communities perceiving that abortions are linked to promiscuity or engaging in sex work or prostitution [9]. Therefore, women who report higher levels of stigma may be more likely to report lower levels of person-centered care because they generally perceive low support, even from trusted individuals such as healthcare providers or family members. Moreover, women with higher levels of stigma may also interact and engage with providers differently, affecting feelings of self-efficacy to demand or expect better care. Additionally, stigma is associated with psychological consequences, including anxiety, depression, and increased physiological distress [26]. In juxtaposition to self-rated health, stigma may therefore be associated with other anxieties related to their health that would contribute to women reporting worse experiences.

This study has a number of limitations. First, women were interviewed at the health facility. Past studies on quality of care find that interviewing women at the facility, directly after their health procedure, is associated with higher levels of satisfaction with care and perceived quality of care [27,28]. Therefore, this may bias our results positively and underestimate poor treatment. Second, this study includes individuals who have taken their first pill at the health facility as well as women who are coming back for their second pill for medication abortion. There may be differences in the type of care and interactions that women receiving their first vs. second pill will have. However, most pain is experienced after the second MA pill and therefore, these differences may be minimal. This study is also limited by a small sample size. While this study is powered to detect differences across two means, it may have lower power to detect differences across covariates and the outcome of interest.

Moreover, small sample size limits our ability to conduct sub-analyses by sub-domains of person-centered care. Future studies should include a larger sample size in order to model the outcome in different ways for ease of interpretation. We conducted sensitivity analyses on person-centered abortion care as a continuous, categorical, and binary outcome. We decided to use the categorical outcome because of ease of interpretation and main results for the categorical outcome are in line with findings for bivariate results for the continuous outcome and bivariate and multivariate results for the binary outcome. Additional covariates were comparable across the various constructions of person-centered abortion care outcomes. The categorical outcome may be more intuitive for health care professionals interested in improving women’s experiences of care compared to a continuous outcome where it may be less clear what a one point increase in person-centered care may translate into for clinical practices; on the other hand, a categorical outcome retains more nuanced information compared to a binary outcome.

Another limitation is that the study only included women from Nairobi County, which comprises an urban population living in both formal and informal settings. Therefore, the findings may not be generalizable to rural communities, as the levels of social support, experiences and socio-economic situations of women in rural areas may be quite different. Women accessing medication abortion pills from less formal settings (i.e. private informal providers) may have quite different experiences from the women in this study as well. Women seek safe abortion services from a variety of facilities, including public and private, and higher and lower level facilities [1]. Given the perception that private facilities are oftentimes of higher quality, it is possible that this sample represents a higher socioeconomic status group compared to those going to lower level public facilities. Future studies should examine person-centered care across a variety of health facilities and contexts.

Our findings highlight the need for greater attention to patient experiences, patient-provider interaction, and an appreciation among abortion providing agencies of how cost, stigma, and method of choice may interact to affect the experiences of women seeking abortion services. Person-centered care requires a reconciliation of the patients’ and doctor's agenda via attention to communication, power and patient autonomy. There are a number of important changes which need to be made. Providers should make the abortion experience for women personal by ensuring the use of names, both the clients and service providers. Such an approach will further de-stigmatize abortion among women. Our findings suggest the value of introducing provider training on medication abortion that is equivalent to the “vocal local” training given to surgical abortion providers and emphasizing greater personalized care.

The lower PCAC scores among women who received medication abortion has broader policy implications because of the global trend in middle-income countries away from surgeries; a trend accelerating in many countries because of growth in over-the-counter sales from pharmacies. Where this is happening, it is likely to further reduce person-centered care for women, as their interactions with providers are reduced to engagement centered on pharmaceutical purchasing. The response to this will need to involve a mix of both policies and programs, increasing non-traditional ways of facilitating significant engagement with women outside of clinical settings. Web and hotline based support networks, outreach workers, and trainings for a wide range of providers of clinical, pharmaceutical, and social support services are likely to all play a role [29–31]. Experts in the field are only recently collaborating to work toward better measures [5,32]. Further efforts are needed to continue to improve women’s quality of care for abortion services across the globe, particularly around women’s experiences of care.

## Supporting information

S1 Table Items for person-centered abortion care scale in Kenya. (DOCX)

S2 Table Distribution of respectful and supportive care sub-scale items stratified by abortion procedure type. (DOCX)

S3 Table Distribution of communication and autonomy sub-scale items stratified by abortion procedure type. (DOCX)
