# Supplementary material for: Is there a difference in women’s experiences of care with medication vs. manual vacuum aspiration abortions? Determinants of person-centered care for abortion services
Source: PLoS One. 2019 Nov 25;14(11):e0225333. doi: 10.1371/journal.pone.0225333 (PMC6876888; doi:10.1371/journal.pone.0225333)
Supplement: S1 Table — (DOCX) [file pone.0225333.s001.docx]

**S1 Table. Items for person-centered abortion care scale in Kenya**

| **Original Domain** | **Question** | **Referred to in text as:** | **Comment** |
| --- | --- | --- | --- |
| Dignity/Respect | How did you feel about the amount of time you waited? | Time to care | Retained for both |
| Dignity/Respect | During your time in this clinic did the doctors, nurses, or other health care providers introduce themselves to you when they first came to see you? | Introduce self | Retained for both |
| Dignity/Respect | Did the doctors, nurses, or other health care providers call you by your name? | Called by name | Retained for both |
| Dignity/Respect | Did the doctors, nurses, or other staff at the facility treat you with respect? | Treated with respect | Retained for both |
| Dignity/Respect | Did the doctors, nurses, and other staff at the facility treat you in a friendly manner? | Friendly | Retained for both |
| Dignity/Respect | During your time in the health facility, would you say you were treated differently because of any personal attribute… like your age, marital status, number of children, your education, wealth, your connections with the facility, or something like that? | Treated differently | Retained for both |
| Dignity/Respect | Did the doctors, nurses, and other staff at the facility show that they cared about you? | Cared | Retained for both |
| Dignity/Respect | Did you feel the doctors, nurses, or other health providers shouted at you, scolded, insulted, threatened, or talked to you rudely? | Verbal abuse | Retained for both |
| Dignity/Respect | Did you feel like you were treated roughly like pushed, beaten, slapped, pinched, physically restrained, or gagged? | Physical abuse | Retained for both |
| Privacy/Confidentiality | When you were speaking to the doctors, nurses or other staff at the facility, did you feel other people not involved in your care could hear what you were discussing? | Privacy | Retained for both |
| Privacy/Confidentiality | Do you feel like your health information was or will be kept confidential at this facility? | Record confidentiality | Retained for both |
| Autonomy | Did you feel like the doctors, nurses or other staff at the facility involved you in decisions about your abortion care? | Involvement in care | Retained for both |
| Autonomy | Did the doctors, nurses or other staff at the facility ask your permission/consent before doing procedures on you? | Consent before procedures | Retained for both |
| Communication | Did the doctors and nurses explain to you why they were doing examinations or procedures on you? | Explain exams | Retained for both |
| Communication | Did the doctors and nurses explain to you why they were giving you any medicine, including pain medicine or medicine to start an abortion? | Explain medicines | Retained for both |
| Communication | Did you feel you could ask the doctors, nurses or other staff at the facility any questions you had? | Ask questions | Retained for both |
| Supportive Care | Did the doctors and nurses at the facility talk to you about how you were feeling? | Ask about feeling | Retained for both |
| Supportive Care | Did the doctors and nurses ask how much pain you were in? | Ask about pain | Retained for both |
| Supportive Care | Do you feel the doctors or nurses did everything they could to help control your pain? | Pain medication given | Retained among SA sub-group; Deleted among MA sub-group |
| Supportive Care | Did you feel the doctors and nurses paid attention to you during your stay in the facility? | Paid attention | Retained for both |
| Supportive Care | Do you think there was enough health staff in the facility to care for you? | Enough staff | Retained for both |
| Supportive Care | Did you feel the doctors, nurses or other staff at the facility took the best care of you? | Took best care | Retained for both |
| Supportive Care | Did you feel you could completely trust the doctors, nurses or other staff at the facility with regards to your care? | Trust | Retained for both |
| Supportive Care | In general, did you feel safe in the health facility? | Safe | Retained for both |

Adapted from Afulani et al, 2017 (Afulani et al., 2017)
