# Supplementary material for: Is there a difference in women’s experiences of care with medication vs. manual vacuum aspiration abortions? Determinants of person-centered care for abortion services
Source: PLoS One. 2019 Nov 25;14(11):e0225333. doi: 10.1371/journal.pone.0225333 (PMC6876888; doi:10.1371/journal.pone.0225333)
Supplement: S2 Table — (DOCX) [file pone.0225333.s002.docx]

**S2 Table. Distribution of respectful and supportive care sub-scale items stratified by abortion procedure type**

| ***Item*** | **N (%)** | | **P-value*** |
| --- | --- | --- | --- |
|  | **Surgical Abortion (N=157)** | **Medication Abortion**  **(N=196)** |  |
| How did you feel about the amount of time you waited? |  |  | 0.649 |
| 0 - Very long | 12 (7.6) | 14 (7.1) |  |
| 1- Somewhat long | 24 (15.3) | 35 (17.9) |  |
| 2 - A little long | 66 (42.0) | 90 (45.9) |  |
| 3 - Very short | 55 (35.0) | 57 (29.1) |  |
| Did the doctors, nurses, or other staff at the facility treat you with respect? |  |  | 0.262 |
| 0 - Never | 0 (0.0) | 2 (1.0) |  |
| 1 - Yes, a few times | 1 (0.6) | 5 (2.6) |  |
| 2 - Yes, most of the time | 17 (10.8) | 25 (12.8) |  |
| 3 - Yes, all of the time | 139 (88.5) | 164 (83.7) |  |
| Did the doctors, nurses, and other staff at the facility treat you in a friendly manner? |  |  | 0.003 |
| 0 - Never | 0 (0.0) | 3 (1.5) |  |
| 1 - Yes, a few times | 1 (0.6) | 0 (0.0) |  |
| 2 - Yes, most of the time | 4 (2.6) | 23 (11.7) |  |
| 3 - Yes, all of the time | 152 (96.8) | 170 (86.7) |  |
| During your time in the health facility, would you say you were treated differently because of any personal attribute… like your age, marital status, number of children, your education, wealth, your connections with the facility, or something like that? |  |  | 0.325 |
| 0 - Yes, all of the time | 2 (1.3) | 0 (0.0) |  |
| 1 - Yes, most of the time | 0 (0.0) | 1 (0.5) |  |
| 2 - Yes, a few times | 6 (3.8) | 6 (3.1) |  |
| 3 - Never | 149 (94.9) | 189 (96.4) |  |
| Did the doctors, nurses, and other staff at the facility show that they cared about you? |  |  | 0.545 |
| 0 - Never | 2 (1.3) | 5 (2.6) |  |
| 1 - Yes, a few times | 3 (1.9) | 5 (2.6) |  |
| 2 - Yes, most of the time | 21 (13.4) | 34 (17.4) |  |
| 3 - Yes, all of the time | 131 (83.4) | 152 (77.6) |  |
| When you were speaking to the doctors, nurses or other staff at the facility, did you feel other people not involved in your care could hear what you were discussing? |  |  | 0.479 |
| 0 - Yes, all of the time | 10 (6.4) | 9 (4.6) |  |
| 1 - Yes, most of the time | 6 (3.8) | 3 (1.5) |  |
| 2 - Yes, a few times | 5 (3.2) | 7 (3.6) |  |
| 3 - Never | 136 (86.6) | 177 (90.3) |  |
| Do you feel like your health information was or will be kept confidential at this facility? |  |  | 0.628 |
| 0 - Never | 3 (1.9) | 6 (3.1) |  |
| 1 - Yes, a few times | 7 (4.5) | 11 (5.6) |  |
| 2 - Yes, most of the time | 21 (13.4) | 33 (16.8) |  |
| 3 - Yes, all of the time | 126 (80.3) | 146 (74.5) |  |
| Do you feel the doctors or nurses did everything they could to help control your pain? |  |  | NA |
| 0 - Never | 14 (8.9) | NA |  |
| 1 - Yes, a few times | 9 (5.7) | NA |  |
| 2 - Yes, most of the time | 16 (10.2) | NA |  |
| 3 - Yes, all of the time | 118 (75.2) | NA |  |
| Did you feel the doctors and nurses paid attention to you during your stay in the facility? |  |  | 0.555 |
| 0 - Never | 2 (1.3) | 4 (2.0) |  |
| 1 - Yes, a few times | 7 (4.5) | 5 (2.6) |  |
| 2 - Yes, most of the time | 25 (15.9) | 39 (19.9) |  |
| 3 - Yes, all of the time | 123 (78.3) | 148 (75.5) |  |
| Did you feel the doctors, nurses, or other health providers shouted at you, scolded, insulted, threatened, or talked to you rudely? |  |  | 0.687 |
| 0 - Yes, all the time | 2 (1.3) | 1 (0.5) |  |
| 1 - Yes, most of the time | 0 (0.0) | 0 (0.0) |  |
| 2 -Yes, a few times | 1 (0.6) | 2 (1.0) |  |
| 3 - No, never | 154 (98.1) | 193 (98.5) |  |
| Did you feel like you were treated roughly like pushed, beaten, slapped, pinched, physically restrained, or gagged? |  |  | 0.619 |
| 0 - Yes, all the time | 1 (0.6) | 2 (1.0) |  |
| 1 - Yes, most of the time | 0 (0.0) | 0 (0.0) |  |
| 2 -Yes, a few times | 0 (0.0) | 1 (0.5) |  |
| 3 - No, never | 156 (99.4) | 193 (98.5) |  |
| Do you think there was enough health staff in the facility to care for you? |  |  | 0.677 |
| 0 - Never | 6 (3.8) | 11 (5.6) |  |
| 1 - Yes, a few times | 13 (8.3) | 13 (6.6) |  |
| 2 - Yes, most of the time | 21 (13.4) | 32 (16.3) |  |
| 3 - Yes, all of the time | 117 (74.5) | 140 (71.4) |  |
| Did you feel the doctors, nurses or other staff at the facility took the best care of you? |  |  | 0.873 |
| 0 - Never | 3 (1.9) | 2 (1.0) |  |
| 1 - Yes, a few times | 1 (0.6) | 2 (1.0 |  |
| 2 - Yes, most of the time | 31 (19.8) | 41 (20.9) |  |
| 3 - Yes, all of the time | 122 (77.7) | 151 (77.0) |  |
| Did you feel you could completely trust the doctors, nurses or other staff at the facility with regards to your care? |  |  | 0.884 |
| 0 - Never | 1 (0.6) | 3 (1.5) |  |
| 1 - Yes, a few times | 3 (1.9) | 4 (2.0) |  |
| 2 - Yes, most of the time | 29 (18.5) | 37 (18.9) |  |
| 3 - Yes, all of the time | 124 (79.0) | 152 (77.6) |  |
| In general, did you feel safe in the health facility? |  |  | 0.450 |
| 0 - Never | 0 (0.0) | 0 (0.0) |  |
| 1 - Yes, a few times | 2 (1.3) | 1 (0.5) |  |
| 2 - Yes, most of the time | 16 (10.2) | 27 (13.8) |  |
| 3 - Yes, all of the time | 139 (88.5) | 168 (85.7) |  |
| *Pearson chi-square test of group differences Notes: NA = Not applicable (this item was not included in the scale among the medication abortion sample). | | | |
