# Supplementary material for: Is there a difference in women’s experiences of care with medication vs. manual vacuum aspiration abortions? Determinants of person-centered care for abortion services
Source: PLoS One. 2019 Nov 25;14(11):e0225333. doi: 10.1371/journal.pone.0225333 (PMC6876888; doi:10.1371/journal.pone.0225333)
Supplement: S3 Table — (DOCX) [file pone.0225333.s003.docx]

**S3 Table. Distribution of communication and autonomy sub-scale items stratified by abortion procedure type**

| ***Item*** | **N (%)** | | **P-value*** |
| --- | --- | --- | --- |
|  | **Surgical Abortion (N=157)** | **Medication Abortion**  **(N=196)** |  |
| During your time in this MSK clinic did the doctors, nurses, or other health care providers introduce themselves to you when they first came to see you? |  |  | 0.788 |
| 0 - No, none of them | 68 (43.3) | 84 (42.9) |  |
| 1 - Yes, a few of them | 39 (24.8) | 56 (28.6) |  |
| 2 - Yes, most of them | 18 (11.5) | 23 (11.7) |  |
| 3 - Yes, all of them | 32 (20.4) | 33 (16.8) |  |
| Did the doctors, nurses, or other health care providers call you by your name? |  |  | 0.022 |
| 0 - Never | 8 (5.1) | 20 (10.2) |  |
| 1 - Yes, a few times | 34 (21.7) | 26 (13.3) |  |
| 2 - Yes, most of the time | 19 (12.1) | 38 (19.4) |  |
| 3 - Yes, all of the time | 96 (61.2) | 112 (57.1) |  |
| Did you feel like the doctors, nurses or other staff at the facility involved you in decisions about your abortion care? |  |  | 0.258 |
| 0 - Never | 23 (14.7) | 44 (22.5) |  |
| 1 - Yes, a few times | 13 (8.3) | 11 (5.6) |  |
| 2 - Yes, most of the time | 17 (10.8) | 19 (9.7) |  |
| 3 - Yes, all of the time | 104 (66.2) | 122 (62.2) |  |
| Did the doctors, nurses or other staff at the facility ask your permission/consent before doing procedures on you? |  |  | 0.470 |
| 0 - Never | 11 (7.0) | 21 (10.7) |  |
| 1 - Yes, a few times | 3 (1.9) | 7 (3.6) |  |
| 2 - Yes, most of the time | 22 (14.0) | 24 (12.2) |  |
| 3 - Yes, all of the time | 121 (77.1) | 144 (73.5) |  |
| Did the doctors and nurses explain to you why they were doing examinations or procedures on you? |  |  | 0.842 |
| 0 - Never | 12 (7.6) | 19 (9.7) |  |
| 1 - Yes, a few times | 4 (2.6) | 6 (3.1) |  |
| 2 - Yes, most of the time | 17 (10.8) | 24 (12.2) |  |
| 3 - Yes, all of the time | 124 (79.0) | 147 (75.0) |  |
| Did the doctors and nurses explain to you why they were giving you any medicine, including pain medicine or medicine to start an abortion? |  |  | 0.252 |
| 0 - Never | 10 (6.4) | 6 (3.1) |  |
| 1 - Yes, a few times | 5 (3.2) | 5 (2.6) |  |
| 2 - Yes, most of the time | 12 (7.6) | 9 (4.6) |  |
| 3 - Yes, all of the time | 130 (82.8) | 176 (89.8) |  |
| Did the doctors and nurses at the facility talk to you about how you were feeling? |  |  | <0.001 |
| 0 - Never | 19 (12.1) | 62 (31.6) |  |
| 1 - Yes, a few times | 11 (7.0) | 20 (10.2) |  |
| 2 - Yes, most of the time | 33 (21.0) | 34 (17.4) |  |
| 3 - Yes, all of the time | 94 (59.9) | 80 (40.8) |  |
| Did you feel you could ask the doctors, nurses or other staff at the facility any questions you had? |  |  | 0.846 |
| 0 - Never | 4 (2.6) | 7 (3.6) |  |
| 1 - Yes, a few times | 10 (6.4) | 9 (4.6) |  |
| 2 - Yes, most of the time | 21 (13.4) | 26 (13.3) |  |
| 3 - Yes, all of the time | 122 (77.7) | 154 (78.67) |  |
| Did the doctors and nurses ask how much pain you were in? |  |  | <0.001 |
| 0 - Never | 19 (12.1) | 93 (47.5) |  |
| 1 - Yes, a few times | 20 (12.7) | 20 (10.2) |  |
| 2 - Yes, most of the time | 29 (18.5) | 18 (9.2) |  |
| 3 - Yes, all of the time | 89 (56.7) | 65 (33.2) |  |
| *Pearson chi-square test of group differences Notes: NA = Not applicable (this item was not included in the scale among the medication abortion sample). | | | |
